# Experiments and Observations on the Contents of the Medullary Cells in Dropsy

**Published:** 1786

**Authors:** John Hall

**Affiliations:** Member of the Corporation of Surgeons, and Teacher of Anatomy in London


### VIII. *Experiments and Obſervations on the Contents of the Medullary Cells in Dropſy. Communicated in a Letter to Dr.* Simmons *by Mr.* John Hall, *Member of the Corporation of Surgeons, and Teacher of Anatomy in London.*

THE following experiments were made to confirm certain opinions concerning the nature and uſe of the marrow, which, however well founded in reaſon, appeared to me to want the aſſiſtance of facts to fix them on a ſtable foundation. This conſideration led me to ſuppoſe that the following facts might prove acceptable to ſome inquirers, in their inveſtigations of this ſubject.

Theſe experiments were made in the Middleſex hoſpital, on a woman who died in September, 1785, after having laboured under an aſcites for ſeveral months. Every particle of adeps appeared to have been conſumed by the long continuance of the diſeaſe. The medulla employed for theſe examinations was taken from the middle of the bodies of the os femoris and os brachii, where we uſually find the moſt pure and adipoſe marrow.

The experiments have been repeated a few days ago, with the ſame conſequences, on a dropfical fubject, brought to the theatre for diffection. In both thefe cafes the contents of the bones was a tranfparent, gelatinous fubftance, fimilar, in appearance, to painter's fize.

### Experiment I.

A tea-fpoonful of the marrow, laid on a card, was expofed to fire, fo as to burn the paper. No inflammation was excited in the marrow, but, after bubbling for fome time, like fkim-milk cheefe, it was dried up into an horny fubftance.

### Experiment II.

A portion of the medulla was fpread on a white porous paper. The paper was doubled together on its moiftened furface, and dried by the fire: the paper was glued together, without the leaft appearance of oily particles, the paper retaining its original opacity.

### Experiment III.

Half a drachm of the marrow was triturated with two ounces of pure cold water; a perfect folution was made, the mixture poffeffing a confiderable degree of tranfparency.

### Experiment IV.

To the above ſolution were added two drachms of olive oil. By agitation, they became perfectly united, and formed a fluid reſembling a mixture of oil and mucilage of gum arabic.

From theſe experiments it appears, that, inſtead of a pure oil in the medullary cells, a mucilaginous ſubſtance, totally devoid of oil, was found to occupy thoſe interſtices. Hence it is evident, that the marrow, as well as the fat, is abſorbed in certain diſeaſes, and probably in all caſes where the blood is not ſufficiently ſupplied with nutritious particles, from whatever cauſe this defect may ariſe; and that the uſe of the marrow is the ſame as that of the fat, under theſe circumſtances, being a ſtorehouſe of nouriſhment for extraordinary occaſions.

The bones in theſe ſubjects were of a very firm texture, ſtrongly reſiſting the action of the ſaw and hammer, which contradicts the opinion entertained by many, that the chief uſe of the marrow is to prevent fragility of bones.

The ſynovia was likewiſe found in a perfect ſtate; and from hence we are aſſured that the marrow is not eſſential to the formation of this lubricating fluid.

I am inclined to think, that, in caſes of univerſal anarſarca, the medullary cells are filled with water, in like manner as the reticular interſtices of the cellular membrane; and I am the more ſtrongly perſuaded of the poſſibility of this, from having ſeen the thigh bone of an ox filled with a pellucid aqueous fluid inſtead of marrow. The truth or fallacy of this ſuppoſition I will endeavour to prove the firſt opportunity.

*Broad Street,*
March 22, 1786.

